# The Role of the Depressor Nasi Septi Muscle in Nasal Air Flow

**DOI:** 10.1007/s00266-020-01693-3

**Published:** 2020-04-03

**Authors:** Ali Seyed Resuli, Fatih Oktem, Sureyya Ataus

**Affiliations:** 1 Department of ENT, Faculty of Medicine, İstanbul Yeni Yüzyıl University, İstanbul, Turkey; 2 Department of ENT, Cerrahpaşa Faculty of Medicine, İstanbul University, İstanbul, Turkey; 3 Department of Neurology, Bahat Hospital, Eski Edirne Asfaltı No: 653 Bahat Hastanesi, Sultangazi, İstanbul, Turkey

**Keywords:** Dermocartilaginous ligament, Musculus depressor septi nasi, Rhinoplasty, Pitanguy’s ligament

## Abstract

**Background:**

Musculus depressor septi nasi and its tendon, the dermocartilaginous ligament, play an important role in external nasal valve and nasal respiration. If the ligament is cut during septorhinoplasty operations, nasal functions of the nose and facial expressions are affected. Therefore, the aim of this study was to investigate the role of M. depressor septi nasi in nasal respiration at open rhinoplasty operations using rhinomanometry and electromyography.

**Methods:**

The study included 29 patients who had only external nasal deformity (nasal hump deformity). All patients underwent open rhinoplasty. The dermocartilaginous ligament of the patients in the study group (DCL + group) was repaired but not in the control group (DCL − group). Rhinomanometry and electromyography were applied to all patients preoperatively and postoperatively.

**Results:**

In the DCL − group, right and left nasal airflow values were significantly lower in post-op (562.92 cm^3^/s and 548.57 cm^3^/s), whereas right, left, and total nasal resistances were significantly lower in pre-op (0.28 Pa/cm^3^/s, 0.22 Pa/cm^3^/s, and 0.11 Pa/cm^3^/s). Statistically significant differences were not found between rhinomanometric measurements in pre-op and post-op values of the DCL + group. Post-op right, left and mean values of M. depressor septi nasi amplitude in the DCL + group (2.05 mV, 2.0 mV, 2.02 mV) were significantly higher than those in the DCL − group (1.52 mV, 1.61 mV, 1.57 mV).

**Conclusion:**

Repair of the dermocartilaginous ligament during open rhinoplasty operations enhances nasal respiratory functions by expanding the external nasal valve through M. depressor septi nasi and allows the nose to participate in mimic movements.

**Level of Evidence IV:**

This journal requires that authors assign a level of evidence to each article. For a full description of these Evidence-Based Medicine ratings, please refer to the Table of Contents or the online Instructions to Authors www.springer.com/00266.

**Electronic supplementary material:**

The online version of this article (10.1007/s00266-020-01693-3) contains supplementary material, which is available to authorized users.

## Introduction

Nasal obstruction has a negative effect on quality of life. The internal nasal valve (INV) is the narrowest part of the nose, and the external nasal valve (ENV) is the entrance to the nose. Although there have been many studies on the INV, there have been few reports regarding the ENV. Nasal muscles with important effects on the INV and ENV are divided into two groups, i.e., the intrinsic muscles (the nasalis, dilator naris anterior, procerus, and depressor septi nasi muscles) that are entirely within the nose, and extrinsic muscles (the levator labii superioris, zygomaticus minor, and orbicularis oris muscles), the fibers of which project out from the nose [1].

**Fig. 1:**
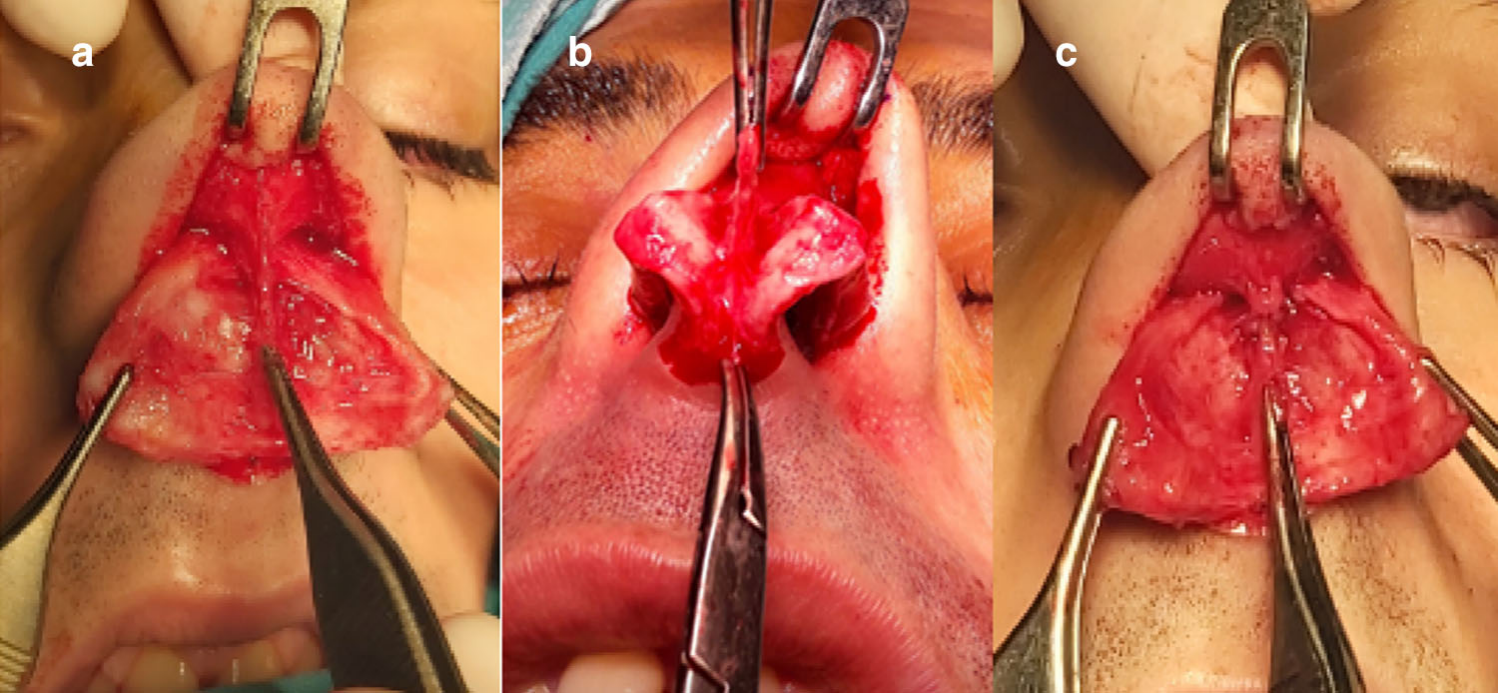
**a** Dermocartilaginous ligament (DCL). **b** DCL-(non-sutured). **c** DCL + (sutured with 6–0 polypropylene)

The facial region differs from other body regions in a number of ways. The soft tissue between the skin and osseocartilaginous skeleton in the face consists of four layers, i.e., the superficial fatty layer, superficial musculoaponeurotic system (SMAS), deep fatty layer, and perichondrium or periosteum layer [2]. The SMAS contains a superficial fatty layer, a fibromuscular layer, a deep fatty layer, a fibrous sheet, and an intercrural ligament. The deep fatty layer composed of loose areolar fat separates the fibromuscular layer from the perichondrium or periosteum. This layer, which has no fibrous reticulations, promotes mobility of the SMAS and thus allows for facial expressions [2, 3]. The fibrous part of the SMAS generally consists of two layers, i.e., the superficial and deep fatty layers. Nasal muscles and their fasciae work as a single component. Furthermore, the SMAS allows for distribution of the forces that result from contractions of the multiple muscles connecting the nasal muscles (and their fascia) to each other and balances their movements. This complex structure comprising the SMAS, nasal muscles, and ligaments allows for the control of phonation, respiration, and facial expressions [4]. The SMAS lies superior to the galea aponeurotica at the rhinion level and inferior to the procerus muscle fascia, moves caudally at the nasal dorsum, and joins with interdomal cross-fibers. The SMAS forms a dermocartilaginous ligament (DCL), as described by Pitanguy, at the supra-tip region, and merges with the depressor septi nasi muscle before passing through the intermediate crural region [5] (Fig. 1a).

The depressor septi nasi muscle originates from the incisive fossa of the maxilla and anterior nasal spine and extends into the maxillary periosteum. The M. depressor septi nasi is associated with the DCL [6, 7]. Some fibers of the muscle project from the upper part of the orbicularis oris muscle. The depressor septi nasi muscle inserts into the columella, membranous septum, and the base of the medial crura, and some fibers also insert into the nasal tip via the medial crura [8]. Some fibers originating from the orbicularis oris muscle lift the central lip while depressing the nasal tip [9]. The depressor septi nasi muscle pulls down the columella, nasal tip, and dorsal margin of the nostril (thus widening the nostril) [10, 11]. The fibers of the depressor septi nasi muscle that project to the nasal tip may contribute to its descending and ascending movements, creating a hypermobile nasal tip during speech and laughter [6, 10, 12]. The depressor septi nasi muscle is also important during nasal respiration; the muscle reduces nasal air resistance by increasing air intake at the nasal vestibular flaps and stretching the membranous septum at the beginning of nasal inspiration [13]. It also contributes dynamically and statically to nasal tip droop and upper lip shortening [14, 15].

The nose has a dynamic structure, to which the nasal muscles make a major contribution. Nasal dynamics should be taken into consideration when seeking to repair visual deformities during open and closed rhinoplasty, due to the role of the nasal muscles in phonation, respiration, and facial expressions [10]. Although there have also been many studies of the effects of DCL and the depressor septi nasi muscle on anatomical appearance, especially that of the nasal tip and upper lip, their effects on the ENV and nasal respiration have not been investigated in detail. During open rhinoplasty, when the DCL is cut, the depressor septi nasi muscle to which it is attached atrophies, as reflected in reduced electrical activity on electromyography (EMG). Therefore, widening of the nostril decreases and nasal resistance increases [16]. This study investigated the effects of anatomical structure on nasal respiration using EMG and rhinomanometry during open rhinoplasty.

## Materials and Methods

### Preparation

This prospective randomized study was performed at the Otorhinolaryngology Clinics of Yeni Yüzyil University and Bahat Hospital in Istanbul, Turkey. A total of 29 patients (18 males and 11 females) with external nasal deformity (nasal hump deformity) and no complaints of nasal obstruction were enrolled in this study. Power analysis indicated that 14 patients were required for each study group (5% type I error level and 95% power). The study was approved by Yeni Yüzyil University Ethics Committee (07.02.2019/002), and written informed consent was obtained from all patients after they had received a thorough explanation of the procedure.

**Fig. 2:**
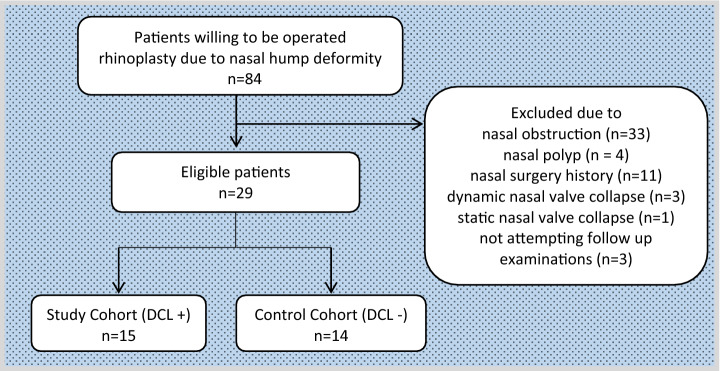
Patient inclusion and exclusion flowchart

The inclusion criteria were age > 18 years and external nasal deformity (nasal hump deformity). To evaluate the effects of DCL and the depressor septi nasi muscle on nasal respiration, only patients eligible for nasal hump surgery were selected from among all patients that applied to our clinic. The exclusion criteria included nasal obstruction, acute or chronic sinusitis, nasal polyps, facial paralysis, myopathies, nasal surgery prior to the study, dynamic nasal valve collapse (as determined by detailed nasal endoscopic examination), and static nasal valve collapse (as determined by the modified Cottle maneuver). The inclusion and exclusion flowchart is shown in Fig. 2.

All patients were randomized into two groups: the DCL + group (15 patients whose DCL was repaired after cutting during rhinoplasty) and the DCL − (control) group (14 patients whose DCL was not repaired). A table of random numbers was used to randomly assign patients to the groups.

### Surgical Technique

Open rhinoplasty was performed by an otolaryngology specialist working in Bahat Hospital and Istanbul Yeni Yüzyil University. Care was taken to avoid damage to the facial nerve branches and nasal muscles during rhinoplasty by taking a full-thickness nasal flap.

The operation began with a transcolumellar incision in the subcutaneous plane up to the medial crura. Care was taken not to damage the soft tissue on the columellar flap and between the medial crura. The perichondrium of the middle crura was cut with sharp scissors. Elevation was achieved in the subperichondrial plane with the help of elevators. After dissecting to the interdomal region, the perichondrium was cut in the caudal edge of the lower lateral cartilage and included in the skin flap. By continuing the dissection to the lateral crura, the central fibromuscular tissue was reached and the DCL was identified. The ligament was marked with 6–0 sutures after disconnecting it from the septum. Caudal subperichondrial dissection was continued until the frontonasal connection was reached, while remaining in the subperiosteal plane. Facial nerve branches stimulating the nasal muscles were preserved under the SMAS layer. As patients with only nasal hump deformity were included in the study, nasal hump resection and lateral osteotomy were performed by ultrasonic osteotomy without affecting the nasal tip or nostrils. After completing nasal hump resection, the DCL was sutured with 6–0 polypropylene sutures in the DCL + group for DCL repair (Fig. 1c); no repair was performed in the control group (DCL − group) (Fig. 1b). The incisions were closed by suturing to complete the operation.

### Electromyographic Technique

**Fig. 3:**
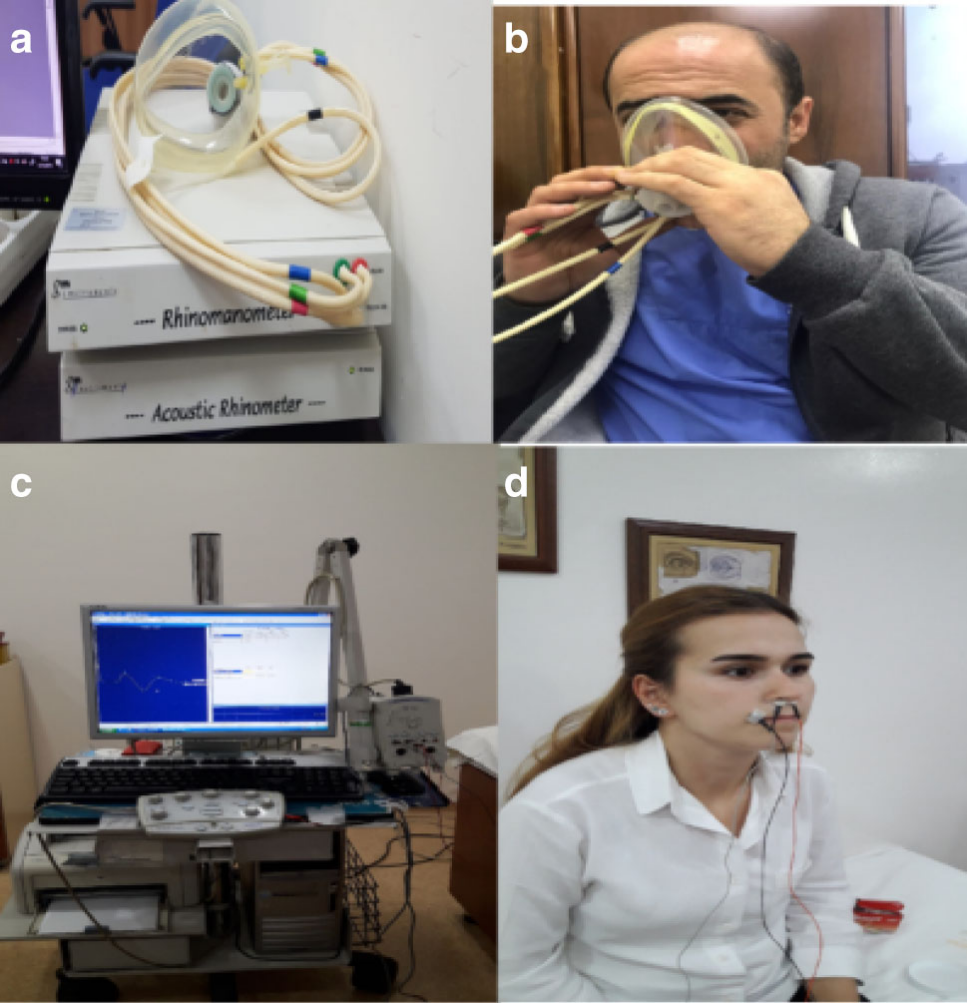
Devices used in the study and their application. **a** Rhinomanometer. **b** Application of the rhinomanometric test. **c** Electromyography. **d** Application of the electromyographic test

EMG was performed in both groups pre- and postoperatively. The lateral branch of the depressor septi nasi muscle widening the nostril was examined bilaterally. Recordings were performed using a Medelec Syrergy N EP-EMG recorder (EP monitoring system + ; Viasys Healthcare, Madison, WI) by a single neurologist. For the analysis, the low filter was set to 500 Hz and the high filter was set to 1,500 Hz. The recordings were bilateral, and the arithmetic means of all amplitudes were calculated. Active electrodes were placed on the junction of the columella and nasal skin, and the reference electrode was placed on the tip of the nose. Recordings were made at rest and during deep nasal inspiration (Fig. 3c, d). The electrical activities of the depressor septi nasi muscle and nasal resistance were analyzed and compared between the groups.

### Rhinomanometric Technique

The patients were escorted to a room maintained at a temperature of 25 °C, with a low noise level, for the rhinomanometry (RMM) measurements after acclimatizing to the hospital environment for 30 min in a sitting position. Xylometazoline hydrochloride (0.01%) was applied twice to both nostrils for decongestion. After placing a nasal probe into one nostril, the patients were asked to breathe through the other nostril with the mouth closed. Transnasal airflow and pressure were recorded by a computer. Nasal airflow resistance was evaluated based on active anterior RMM measurements. All measurements were performed by a single otolaryngologist from the Otorhinolaryngology Department of Cerrahpasa Medical Faculty, Istanbul University, at a steady pressure of 150 Pa, as recommended by the European Rhinomanometry Standardization Committee [17, 18]. Nasal airflow resistance was evaluated based on active anterior RMM measurements. Nasal resistance was determined at a reference pressure of 150 Pa, which was below the maximal 5% coefficient of variation, based on 10 respiratory cycles (inspiration plus expiration) for each nasal cavity. The average pressure differential (P), amount of airflow passing through the nasal cavity (V), and nasal resistance (R) were calculated automatically using the formula $R = P/V$ in Pa/cm^3^/s for each cavity by a microprocessor. Total nasal resistance was determined according to Ohm’s law:

19 $$R_{{{\text{total}}}} = \left( {R_{{{\text{right}}}} \times R_{{{\text{left}}}} } \right)/\left( {R_{{{\text{right}}}} + R_{{{\text{left}}}} } \right).$$

**Fig. 4:**
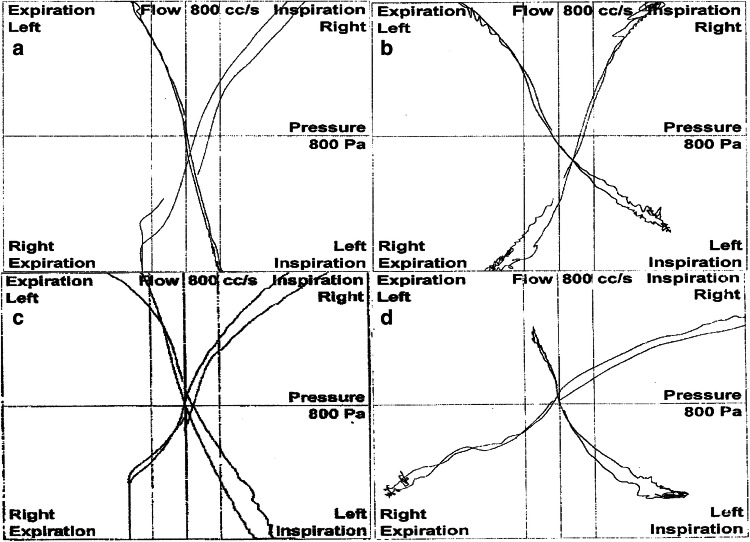
Rhinomanometry (RMM) results. **a** Preoperative RMM results in the dermocartilaginous ligament (DCL)–group. **b** Postoperative RMM results in the DCL − group. **c** Preoperative RMM results in the DCL + group. **d** Postoperative RMM results in the DCL + group

**Fig. 5:**
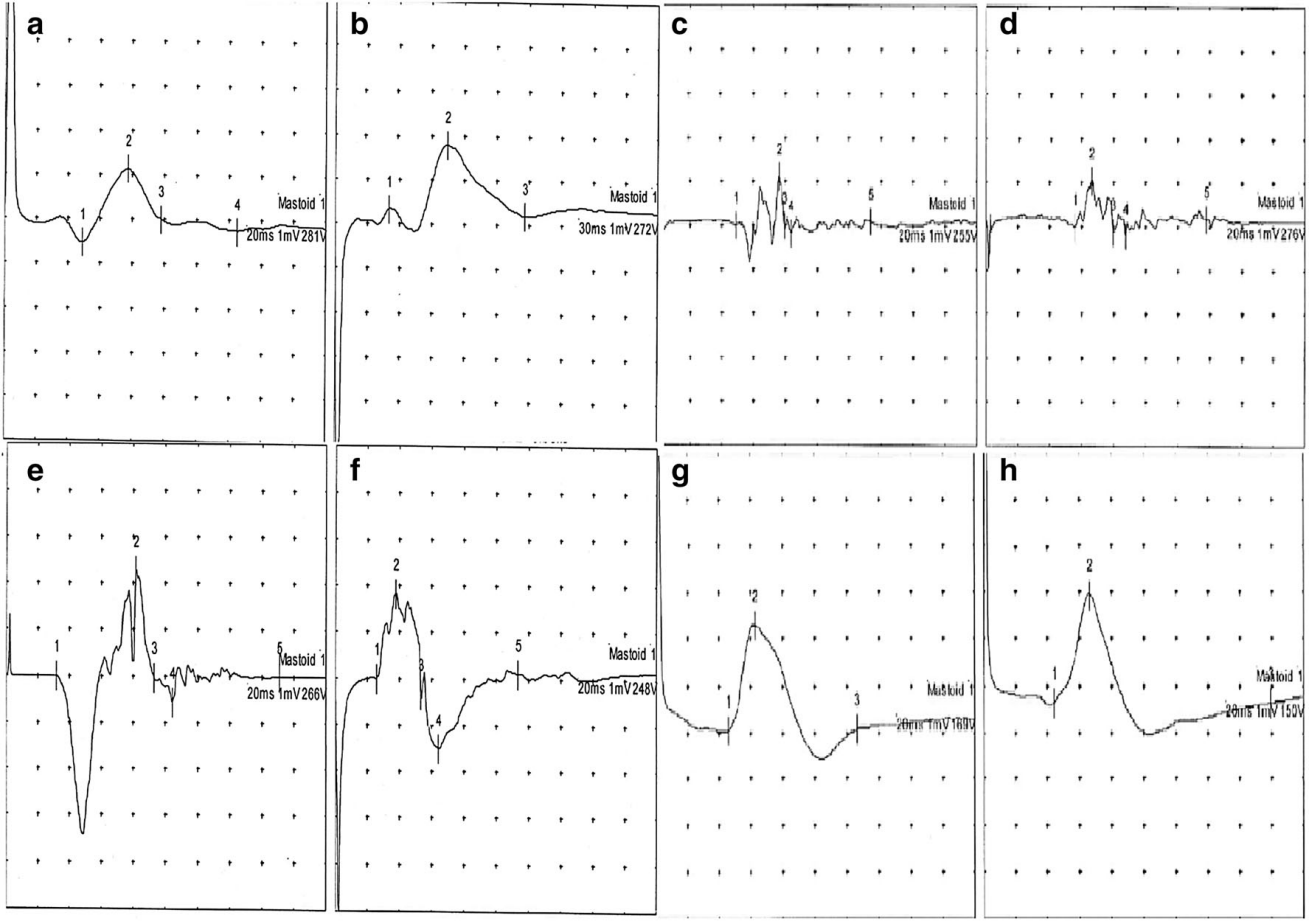
Electromyographic (EMG) results. **a** Preoperative left depressor septi nasi muscle activity in the DCL − group. **b** Preoperative right depressor septi nasi muscle activity in the DCL − group. **c** Postoperative left depressor septi nasi muscle activity in the DCL − group. **d** Postoperative right depressor septi nasi muscle activity in the DCL − group. **e** Preoperative left depressor septi nasi muscle activity in the DCL + group. **f** Preoperative right depressor septi nasi muscle activity in the DCL + group. **g** Postoperative left depressor septi nasi muscle activity in the DCL + group. **h** Postoperative right depressor septi nasi muscle activity in the DCL + group

The right, left, and total airflow and airway resistances were measured. RMM recordings were performed using an NR6 rhinomanometer (GM Instruments Ltd., Kilwinning, UK) (Fig. 3a, b). The specialists who performed the rhinomanometric and myographic measurements were blinded to patient group. RMM was performed twice in all patients (once before the operation and once at 4 months postoperatively) (Fig. 4). EMG was also performed twice in all patients (once before the operation and once between 6 and 7 months postoperatively) (Fig. 5). EMG was performed at least 6 months after the surgery to allow optimal healing in cases where the facial nerve branches were damaged during the operation.

### Statistical Analysis

Statistical analyses were performed using SPSS software (ver. 20; SPSS Inc., Chicago, IL, USA). The data are reported as the mean ± standard deviation (SD). Normality analysis showed that the data were not normally distributed. Therefore, the Mann–Whitney *t* test and Wilcoxon’s test were used to compare the means of independent and dependent variables, respectively. In all analyses, *P* < 0.05 was taken to indicate statistical significance.

## Results

In this study, the DCL was sutured in 15 patients in the DCL + group (10 males and 5 females) undergoing open rhinoplasty surgery due to external nasal deformity (nasal hump deformity), while no suturing of the DCL was performed in 14 patients in the DCL − group (8 males and 6 females). The depressor septi nasi muscle activity, as determined by EMG, was lower in the DCL − group than the DCL + group. As the DCL was cut but not sutured in the DCL − group, the strength of the depressor septi nasi muscle was decreased. Furthermore, airflow and nasal resistance during respiration were also measured by RMM, and the results indicated that nasal resistance was increased in the DCL − group.

The pre- and postoperative RMM frequencies of the groups and the correlations thereof are shown in Table 1. Significant differences were found between the groups in postoperative right nasal airflow, right nasal resistance, and left nasal resistance. The mean postoperative right nasal airflow was 562.92 cm^3^/s in the DCL − group and 653.33 cm^3^/s in the DCL + group. The mean postoperative right nasal resistance was 0.33 Pa/cm^3^/s in the DCL − group and 0.24 Pa/cm^3^/s in DCL + group. The mean postoperative left nasal resistance was 0.29 Pa/cm^3^/s in the DCL − group and 0.26 Pa/cm^3^/s in the DCL + group. In accordance with these data, the patients in the DCL − group began complaining of nasal obstruction from month 2 after surgery.

**Table 1:** Pre- and postoperative RMM frequencies of the two groups and their correlations (Mann–Whitney *U* test, *P* < *0*.*05*)

			DCL − group	DCL + group	*P*
Pre-op	Nasal airflow	Right	593.85 ± 173.08	660.20 ± 168.07	0.43
		Left	638.21 ± 182.20	670.06 ± 143.79	0.74
		Total	1272.21 ± 71.33	1330.26 ± 77.75	0.69
	Nasal resistances	Right	0.28 ± 0.15	0.23 ± 0.06	0.38
		Left	0.22 ± 0.05	0.26 ± 0.15	0.44
		Total	0.11 ± 0.02	0.12 ± 0.03	0.98
Post-op	Nasal airflow	Right	562.92 ± 110.66	653.33 ± 156.20	0.04
		Left	548.57 ± 148.83	663.53 ± 100.10	0.06
		Total	1071.35 ± 66.3	1316.86 ± 61.94	0.02
	Nasal resistances	Right	0.33 ± 0.16	0.24 ± 0.06	0.00
		Left	0.29 ± 0.05	0.26 ± 0.15	0.03
		Total	0.15 ± 0.06	0.91 ± 3.06	0.12

The pre- and postoperative RMM results of the non-sutured group (DCL − group) are shown in Table 2. Significant differences were found between all pre- and postoperative measurements in the DCL − group. Postoperative right, left, and total nasal airflow values were significantly lower (562.92, 548.57, and 1,071.35 cm^3^/s, respectively) than the corresponding preoperative values in the DCL − group (593.85, 638.21, and 1,272.21 cm^3^/s, respectively [*P*-values]). The right, left, and total nasal resistance values were significantly lower preoperatively (0.28, 0.22, and 0.11 Pa/cm^3^/s, respectively) than postoperatively in the DCL − group (0.33, 0.29, and 0.15 Pa/cm^3^/s, respectively [*P*-values]).

**Table 2:** Pre- and postoperative RMM results of the non-sutured group (DCL −) (Wilcoxon’s test, *P* < 0.05)

		Pre-op	Post-op		
Nasal airflow	Right	593.85 ± 173.08	562.92 ± 110.66	− 2.29	0.02
	Left	638.21 ± 182.20	548.57 ± 148.83	− 3.29	0.00
	Total	1272.21 ± 71.33	1071.35 ± 66.3	− 3.23	0.00
Nasal resistances	Right	0.28 ± 0.15	0.33 ± 0.16	− 3.30	0.00
	Left	0.22 ± 0.05	0.29 ± 0.10	− 3.30	0.00
	Total	0.11 ± 0.02	0.15 ± 0.06	− 3.20	0.00

Pre- and postoperative RMM results of the sutured group (DCL + group) are shown in Table 3. The DCL + group showed no significant differences in any of the RMM measurements, pre- versus postoperatively.

**Table 3:** Pre- and postoperative RMM results of the sutured group (DCL +) (Wilcoxon’s test, *P* < 0.05)

		Pre-op	Post-op		
Nasal airflow	Right	660,.20 ± 168.07	653.33 ± 156.20	− 0.34	0.73
	Left	670.06 ± 143.79	663.57 ± 100.10	− 0.22	0.82
	Total	1071.35 ± 66.3	1316.86 ± 61.94	− 0.14	0.88
Nasal resistances	Right	0.23 ± 0.06	0.24 ± 0.06	− 1.15	0.24
	Left	0.26 ± 0.15	0.23 ± 0.03	− 0.11	0.90
	Total	0.12 ± 0.03	0.91 ± 3.06	− 1.20	0.22

The pre- and postoperative EMG activity of the depressor septi nasi muscle activity in both groups and the relationships between them are shown in Table 4. The postoperative EMG results showed significant differences between the two groups, but there were no significant differences in postoperative EMG results. Postoperative right, left, and mean depressor septi nasi muscle amplitudes in the DCL + group (2.05, 2.0, and 2.02 mV, respectively) were significantly higher than those in the DCL − group (1.52, 1.61, and 1.57 mV, respectively [*P*-values]).

**Table 4:** Pre- and postoperative EMG activity of the depressor septi nasi muscle in both groups and their correlations (Mann–Whitney *U* test, *P* < 0.05)

		DCL − group	DCL + group	*P*
Post-op	Right	1.52 ± 0.37	2.05 ± 0.21	0.00
	Left	1.61 ± 0.26	2 ± 0.19	0.00
	Mean of right and left	1.57 ± 0.23	2.02 ± 0.18	0.00
Pre-op	Right	2.11 ± 0.29	2.1 ± 0.19	0.72
	Left	2.12 ± 0.25	2.05 ± 0.18	0.21
	Mean of right and left	2.11 ± 0.23	2.08 ± 0.17	0.46

Correlations between the pre- and postoperative EMG amplitudes in the two groups are shown in Table 5. Significant differences were found between the pre- and postoperative values for all measurements in both groups. The postoperative right, left, and mean EMG amplitudes were significantly lower than the corresponding preoperative values in both the DCL − group (1.52, 1.61, and 1.57 mV vs. 2.11, 2.12, and 2.11 mV, respectively [*P*-values]) and the DCL + group (2.05, 2.0, and 2.02 mV vs. 2.1, 2.05, and 2.08 mV, respectively [*P*-values]).

**Table 5:** Pre- and postoperative EMG results of the two groups and their correlations (Wilcoxon’s test, *P* < 0.05)

		Post-op	Pre-op	Z	*P*
DCL − group	Right	1.52 ± 0.37	2.11 ± 0.29	− 3.32	0.001
	Left	1.61 ± 0.26	2.12 ± 0.25	− 3.32	0.001
	Mean of right and left	1.57 ± 0.23	2.11 ± 0.23	− 3.31	0.001
DCL + group	Right	2.05 ± 0.21	2.1 ± 0.19	− 2.33	0.020
	Left	2 ± 0.19	2.05 ± 0.18	− 2.43	0.015
	Mean of right and left	2.02 ± 0.18	2.08 ± 0.17	− 2.88	0.004

## Discussion

There has been a recent increase in the number of septorhinoplasties performed. In addition to the aesthetic appearance, functional results are important. Compared to other nasal soft tissue layers, the anatomy of the muscular layer is less well understood and is also assigned less importance by rhinoplasty surgeons. In fact, the anatomy and function of the muscular layer, ligaments, and SMAS of the nose have often been disregarded. Due to aesthetic concerns, osseocartilaginous structures have been given more attention in rhinoplastic surgery; however, attention should also be paid to the nasal muscles to achieve better functional and aesthetic results [16].

Nasal congestion is one of the major postoperative complaints of rhinoplasty patients. Recently, studies have begun to investigate how nasal function may be affected by rhinoplasty. However, the effects of rhinoplasty on the ENV have not been investigated in sufficient detail. In addition to achieving the desired shape, size, and position of the nose after rhinoplasty, the nasal air passage must not be damaged.

The functionally important ENV is formed by the caudal edges of the lower lateral cartilage, the alar soft tissue, the membranous septum, and the edge of the nostril. This area is also called the nostril opening area. Nasal muscles play an important role in respiratory function [19]. Intrinsic and extrinsic nasal muscles are involved in inspiration and expiration and in opening the nasal airway. Resistance in the nasal airway originates from the ENV and INV [20]. It has been demonstrated that nasal air resistance arises from the anterior part of the nose and nasal valve [20, 21]. The depressor septi nasi muscle decreases ENV resistance when contracted; the medial part of this muscle depresses the nasal tip, and the lateral part dilates the nostril. The depressor septi nasi muscle originates from the maxilla and fibers projecting from the orbicularis oris muscle and inserts into the columella, membranous septum, base of the medial crura, and nasal tip through the medial crura [8]. The depressor septi nasi muscle extends the airway by pulling the nasal tip and membranous septum; it also narrows the labiocolumellar angle by pulling up the upper lip, which is of aesthetic importance [22].

Although some researchers have downplayed the importance of the nasal muscles, they play a major role in mimic movements and nasal respiration [23, 24]. Nasal muscle involvement in nasal valve collapse has been investigated in many studies using EMG. Aksoy et al. [25] investigated the role of the nasal muscles in nasal valve dysfunction: Patients with dynamic nasal valve collapse were compared to healthy volunteers, and significant nasal muscle abnormalities were found in the valve collapse group. In an RMM study, Kienstra et al. [26] induced paralysis of the intrinsic nasal muscles by lidocaine injection and found that this increased nasal airflow and decreased nasal resistance. Recent studies have shown that even small changes in the diameter of the ENV have significant effects on nasal resistance [21]. In this study, the depressor septi nasi muscle EMG activity was higher in the DCL + group (sutured) than the DCL − group (non-sutured); moreover, left and right nasal resistance was significantly lower postoperatively in the DCL + group than the DCL − group, as shown by RMM.

Nasal airflow and nasal resistance are controlled by blood vessels in the mucosa. Venous sinusoids, especially in the inferior turbinate, are under the control of the autonomic nervous system. Activation of the sympathetic nervous system results in nasal decongestion, while activation of the parasympathetic nervous system causes congestion. Blood vessels in the septum and inferior turbinates are differentiated, which is important in the INV region but not the ENV region [27]. Mucosal congestion, concha hypertrophy, and structural deformity are the most common causes of nasal obstruction. Decongestants allow determination of the contribution of the mucosa to nasal obstruction. Nasal resistance should be measured separately in both nasal cavities before and after use of topical decongestants. Mucosal congestion improves after application of decongestants, except in cases with structural disorders. Total nasal resistance is more valuable for evaluating obstructive symptoms because it is unaffected by the nasal cycle [28–32]. Total nasal resistance is the most valuable RMM measurement, with values between 0.12 and 0.33 Pa/cm^3^/s accepted as normal [33]. In this study, the pre- and postoperative total nasal resistance values in the DCL − group were significantly different, at 0.11 and 0.15, respectively; in the DCL + group, however, there was no significant difference. In addition, patients in the DCL − group began to complain of nasal obstruction during month 2 after the operation.

The role of muscular activity in the nasal airway has been demonstrated previously by various methods [4, 26]. Kienstra et al. [26] reported that RMM can be used to determine the function of the nasal muscles. Bruintjes et al. [4] reported that EMG could be used to selectively record nasal muscle activity. EMG is a highly valuable method for assessing nasal muscle function. Nasal motor unit potentials can be recorded using surface electrodes; moreover, although the nasal muscles are very small, they can be discriminated based on their electrical activities [10, 16, 34]. In this study, a similar method was used, and as shown in Table 4, right, left, and mean depressor septi nasi muscle amplitudes in the DCL + group were significantly higher than those in the DCL − group. Consistent with our work, a previous study indicated that nasal muscle EMG activity and nasal respiration are impaired when the adhesion sites of the nasal muscles are damaged during external rhinoplasty [16]. Although the EMG activities of the internal nasal muscles were within the normal range, reduced depressor septi nasi muscle activity was observed in our DCL − group. These observations suggested that muscular atrophy had occurred, due to a lack of tension in the muscle after cutting the DCL.

The ENV region is very complicated and remains poorly understood. It is influenced by many static and dynamic factors. We explored the relationship between the dynamic structure of the DCL and M. depressor septi nasi, and the ENV and nasal breathing. Many rhinoplastic surgical maneuvers affect the ENV. Surgical procedures employed to treat caudal septal deformities (bringing the septum into the midline via suturing, and placement of a septal batten graft) decrease ENV resistance, as do columellar struts placed to treat nasal tip ptosis, caudal septal extension grafting, caudal septal replacement grafting, and medial crura suturing to the caudal septum[35, 36]. In addition, the ENV is expanded by nasal deprojection; nasal breathing is increased [37]. Strengthening of the lateral nasal walls with batten grafts prevents nasal collapse during deep inspiration. Batten grafts should be placed in the caudal part of the lateral crura to ensure ENV support [38]. Lateral crus modification (correction of cephalic malpositioning of the long axis of the lateral crura and sagittal malpositioning of the short axis) increases the width of the ENV and thus the volume of inspiratory nasal breathing [39]. Alar rim grafts strengthen the nostril margin and increase the nostril cross-section [40]. If nasal tip deprojection cannot be performed, narrowing of the large columella via soft tissue excision and suturing expands the ENV [41]. Suspension suturing is a minimally invasive method used to support the lateral nasal wall. Many such techniques are available. Commonly, cephalic rotation of the lateral crura is increased by permanent suturing of the lateral crura to the pyriform aperture or orbital rim [42, 43].

Patients with positive Cottle tests, narrow nostrils, who require heavy glasses, and/or have thick sebaceous skin, a deep alar groove, the parenthesis tip deformity, weakness of the facial and/or nasal muscles, anterior septal deviation, and/or thin and weak nasal cartilage tissue benefit from DCL protection. In such patients, it is appropriate to add surgical maneuvers that support the ENV. However, patients with thin skin, strong cartilage, a gummy smile, and nasal resistance within the normal limits should not undergo DCL repair [21, 44–47]. We do not recommend DCL repair for patients with prominent nasal humps, especially those with a hypertrophic M. depressor septi nasi; nasal tip droop increases after hump resection [48]. DCL repair should not be performed in patients with a short columella and a hypertrophic M. depressor septi nasi [49]. An increase in M. depressor septi nasi activity causes “smiling deformities.” The DCL should not be preserved in patients with nasal tip droop, upper lip shortening, or a transverse crease in the mid-philtral area; a severe laughing deformity will develop if the DCL is retained [50, 51].

This study had some limitations. First, pre- and postoperative photographs of the patients were not compared in terms of aesthetic outcomes (upper lip length and nostril shape). Assessment of the change in vertical diameter of the nares during inspiration, pre- versus postoperatively, would have facilitated validation of our RMM results. Moreover, the effects of the depressor septi nasi muscle on upper lip length were not taken into consideration. Further studies to examine the respiratory and aesthetic outcomes of DCL suture during rhinoplasty are required. Some subjective complaints of nasal obstruction were made 2 months after operation; the frequency did not differ between the two groups. All surgeries were performed by a single surgeon, perhaps introducing bias.

## Conclusion

Even minimal changes in the ENV have significant effects on nasal resistance. An increase in total air resistance was detected in the DCL − group in the present study; this was attributed to a decrease in depressor septi nasi muscle EMG activity, and an inability to expand the nostrils through activation of this muscle. Furthermore, the EMG results of the depressor septi nasi muscle were normal in the group in which the DCL was sutured, and RMM analysis indicated that the nasal resistance was lower in this group than in the non-sutured group.

Some reports in the literature emphasize that the depressor septi nasi muscle should be cut during rhinoplasty to prevent the nasal tip from being pulled down, especially when laughing, and to prevent a decrease in nasolabial angle during the postoperative period [47, 52]. However, these reports only took aesthetic results into consideration; moreover, RMM and EMG were not performed and the dynamic functions of the nose were not evaluated. Repair of the DCL during open rhinoplastic surgery allows the nose to participate in mimic movements and prevents loss of nasal respiratory function. However, studies investigating both aesthetic and functional outcomes of the depressor septi nasi muscle and DCL are required.

Although studies evaluating the functions of nasal muscles have been conducted, no studies focused specifically on the function of the depressor septi nasi muscle. Thus, additional studies including large numbers of patients are required to validate our results.

## Electronic supplementary material

Below is the link to the electronic supplementary material.

Supplementary file1 (MP4 150607 kb)
